# Diabetes‐Related Disruption of White Matter Microstructural Integrity is Associated with Faster Late‐Life Cognitive Decline

**DOI:** 10.1002/alz.089219

**Published:** 2025-01-09

**Authors:** Han Tong, Ana W. Capuano, Roshni Biswas, Shengwei Zhang, David A. Bennett, Konstantinos Arfanakis, Zoe Arvanitakis

**Affiliations:** ^1^ Rush University Medical Center, Chicago, IL USA; ^2^ Rush Alzheimer's Disease Center, Rush University Medical Center, Chicago, IL USA; ^3^ Rush University, Chicago, IL USA

## Abstract

**Background:**

Type‐2 diabetes mellitus is associated with both altered white matter microstructural integrity and increased risk of dementia. However, whether there is a link between the white matter changes in older adults with diabetes and their cognitive decline remains unclear. The objective of this study was to examine the association of diabetes‐related changes in white‐matter microstructural integrity with longitudinally assessed late‐life cognitive function.

**Method:**

A total of 674 older adults were studied (mean age at time of scan: 78.4 years; 125 males; 104 with diabetes), among participants of an ongoing, community‐based cohort study of aging at the Rush Alzheimer’s Disease Center. Annual clinical evaluations included detailed neuropsychological testing from which a global and five domain‐specific cognitive summary measures were derived (mean follow‐up duration: 3.0 years). High‐quality (3T) brain magnetic resonance imaging scans were acquired, and quality‐assured diffusion tensor imaging data were available for analyses. Tract‐based statistical analysis (TBSS) was used to project each fractional anisotropy (FA) image to the IIT white matter skeleton. Voxel‐wise generalized linear regression models were fitted using Permutation Analysis of Linear Models in FSL, with FA and mean diffusivity (MD) as the outcome and diabetes as predictor, and co‐variates of age, sex, education, scanner type, and white matter hyperintensities. Using linear mixed‐effects models and controlling for age, sex, education, and diabetes, we examined the association between median FA of all significant voxels and longitudinally assessed cognitive function data.

**Result:**

Diabetes was associated with lower FA in the rostral body of the corpus callosum (p<0.05, threshold‐free cluster enhancement, family‐wise error corrected). A lower median FA of these voxels was associated with a faster rate of decline in global cognition (estimate=0.121 [SE=0.056], p=0.030) and three cognitive domains including semantic memory (estimate=0.225 [SE=0.090], p=0.013), working memory (estimate=0.205 [SE=0.081], p=0.012) and visuospatial abilities (estimate=0.193 [SE=0.090], p=0.032), but not episodic memory and perceptual speed. No difference was found in MD between older adults with diabetes and those without.

**Conclusion:**

Diabetes‐related disruption of white matter microstructural integrity in the rostral body of corpus callosum in older adults may be associated with faster late‐life cognitive decline.

